# IFI30 Knockdown Inhibits ESCC Progression by Promoting Apoptosis and Senescence via Activation of JNK and P21/P16 Pathways

**DOI:** 10.1111/1759-7714.70063

**Published:** 2025-04-05

**Authors:** Wenyao Xie, Sisi Wei, Caiting Feng, Yuhui Fu, Zhe Zhang, Suli Dai, Cong Zhang, Lianmei Zhao, Baoen Shan

**Affiliations:** ^1^ Research Center The Fourth Hospital of Hebei Medical University Shijiazhuang China; ^2^ Department of Oncology Handan Central Hospital Handan China; ^3^ Key Laboratory of Tumor Prevention and Precision Diagnosis and Treatment of Hebei, Clinical Oncology Research Center The Fourth Hospital of Hebei Medical University Shijiazhuang Hebei Province China; ^4^ Department of Thoracic Surgery Handan First Hospital Handan China

**Keywords:** apoptosis, ESCC, IFI30, JNK pathway, senescence

## Abstract

**Background:**

Esophageal squamous cell carcinoma (ESCC) is a prevalent and deadly cancer, making it essential to understand the molecular mechanisms influencing its development and prognosis. The role of interferon‐gamma‐inducible protein 30 (IFI30) in antigen processing is well‐established, but its impact on the progression of ESCC remains unclear. This study aimed to investigate the biological function and potential mechanisms of IFI30 in ESCC progression.

**Methods:**

Public databases, proteomics, and immunohistochemistry (IHC) were employed to analyze IFI30 expression. Cell proliferation, migration, and invasion were evaluated using MTS, colony formation, wound healing, and transwell assays. Nude mouse xenograft models were established to assess the effects of IFI30 knockdown in vivo. Quantitative proteomics was utilized to identify differentially expressed proteins (DEPs) and pathways altered by IFI30 knockdown. Cell apoptosis and senescence were evaluated by flow cytometry, SA‐β‐gal staining, and reactive oxygen species (ROS) analysis.

**Results:**

IFI30 was highly expressed in ESCC and was correlated with advanced stage and poor prognosis. IFI30 knockdown inhibited ESCC cell proliferation, migration, and invasion in vitro and suppressed tumor growth in vivo. DEPs were mainly enriched in biological pathways related to apoptosis, mitophagy, cellular senescence, and lysosome. Furthermore, IFI30 knockdown in ESCC cells upregulated HRAS expression, increased ROS production, activated the JNK signaling pathway, and elevated the expression of P16 and P21, thereby promoting apoptosis and senescence.

**Conclusions:**

This study suggests that IFI30 may regulate the JNK and P21/P16 pathways, exerting pro‐tumorigenic effects in ESCC. IFI30 could serve as a potential novel target for ESCC treatment.

## Graphical abstract

IFI30 is highly expressed in ESCC. Knockdown of IFI30 may upregulate HRAS and promote ROS generation, thereby activating the JNK signaling pathway and increasing p16/p21 expression, ultimately inducing apoptosis and senescence in ESCC cells. This study underscores the pivotal role of IFI30 in regulating apoptosis and senescence in ESCC cells and provides a potential new strategy for targeted therapy of ESCC (Drawn by Figdraw, ID: ATRRI64af0).

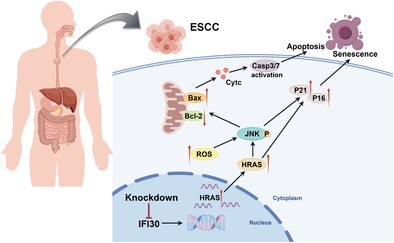

## 1 Introduction

Esophageal cancer is the eighth most prevalent malignancy and the sixth leading cause of cancer‐related mortality worldwide [1]. China exhibits a notably high incidence of esophageal cancer, contributing to nearly half of the global incidence and mortality rates [2]. The common pathological types of esophageal cancer are esophageal squamous cell carcinoma (ESCC) and esophageal adenocarcinoma, with ESCC accounting for 85.79% of all esophageal cancer cases in China [3]. Despite advancements in the diagnosis and treatment of ESCC, which have resulted in improvements in the 5‐year survival rate, the prognosis remains unfavorable due to the fact that the majority of patients are diagnosed at advanced stages. In addition to conventional chemotherapy and radiation therapies, only drugs targeting the vascular endothelial growth factor receptor and programmed cell death receptor 1 immune checkpoint inhibitors have been approved for clinical application. Overall, effective treatments targeting ESCC remain limited, and there is a lack of specific therapeutic targets [4, 5]. Consequently, there is an urgent need to investigate the underlying molecular mechanisms of ESCC progression to identify novel and potentially critical therapeutic targets and prognostic biomarkers.

Interferon‐gamma‐inducible protein 30 (IFI30), an interferon‐stimulated gene, encodes gamma‐interferon‐inducible lysosomal thiol reductase (GILT), which is the sole enzyme known to catalyze the reduction of disulfide bonds in the endocytosis pathway. This enzyme is primarily localized in lysosomes and the cytoplasm [6]. IFI30 is involved in the cross‐presentation pathway of major histocompatibility complex (MHC) class I and the antigen processing pathway of MHC class II in adaptive immunity. It is constitutively expressed in a majority of antigen‐presenting cells, including monocytes, macrophages, dendritic cells, and B cells [7, 8]. Recent research has demonstrated that IFI30 is aberrantly expressed in various cancers, including breast cancer [9, 10], melanoma [11], and glioma [12]. Beyond its well‐established immunological function, IFI30 has been implicated in tumor initiation and progression, reactive oxygen species (ROS) generation, and autophagy activation, making it a potential target for cancer therapy [6]. IFI30 has been documented to exhibit diverse roles across various cancer types. Elevated expression levels of IFI30 have been associated with improved patient survival in several cancers, including diffuse large B‐cell lymphoma [13], gastroesophageal junction adenocarcinoma [14], melanoma [7, 15], and cervical cancer [16]. Conversely, IFI30 expression has shown an inverse relationship with survival outcomes in other malignancies, including glioma [12, 17, 18, 19], prostate cancer [20, 21], and gastric cancer [22]. The role of IFI30 in breast cancer and clear cell renal carcinoma remains controversial [9, 10, 23, 24, 25]. Therefore, further research is warranted to elucidate the function and underlying mechanisms of IFI30 in different cancers, aiming to determine its viability as a diagnostic and prognostic biomarker or as a therapeutic target. However, the specific role and mechanism of IFI30 in the progression of ESCC remain unknown.

This study aims to investigate the role and mechanisms of IFI30 in ESCC progression, providing a theoretical basis for the development of novel diagnostic and prognostic biomarkers, as well as potential therapeutic strategies. Analysis of public databases, proteomics, and immunohistochemistry (IHC) revealed that IFI30 expression is elevated in ESCC tissues and is associated with advanced stage and poor prognosis. IFI30 knockdown in ESCC inhibited cell proliferation, migration, and invasion in vitro and suppressed tumor growth in vivo. Knockdown of IFI30 in ESCC cells increased ROS production, apoptotic protein levels, senescence‐associated β‐galactosidase (SA‐β‐gal) expression, and cell cycle arrest in the G0/G1 phase, suggesting a promotion of apoptosis and senescence. Additionally, IFI30 knockdown upregulated HRAS expression in ESCC cells, activated the c‐Jun N‐terminal kinase (JNK) signaling pathway, and increased the expression of P16 and P21. Taken together, these findings indicate that IFI30 promotes ESCC development and could serve as a potential novel target for its treatment.

## 2 Materials and Methods

### 2.1 Data Gathering and Analysis of Differentially Expressed Genes in Esophageal Cancer

The GEPIA2 database website (http://gepia2.cancer‐pku.cn/), based on the TCGA cohort, was utilized to evaluate the mRNA expression levels of IFI30 and HRAS [26], as well as the correlation between the expression of IFI30 and HRAS in normal tissues and esophageal carcinoma (ESCA) tissues. The correlation between IFI30 mRNA expression levels and tumor staging and grading in normal tissues and ESCA tissues was analyzed using the UALCAN database based on the TCGA cohort. Gene expression profiles (GSE44021) from the GEO database (https://www.ncbi.nlm.nih.gov/geo/), based on the GPL96, GPL570, and GPL571 platforms, were retrieved to profile mRNA expression in 113 paired ESCC samples. Kaplan–Meier survival analysis was performed using the Kaplan–Meier Plotter (http://kmplot.com).

### 2.2 Patient Tissues

This study included three independent clinical cohorts. Cohort 1 consisted of 82 pairs of ESCC and adjacent non‐cancerous tissue samples, which were frozen at −80°C following excision for proteomic analysis. Cohort 2 included 41 pairs of ESCC and adjacent non‐cancerous tissue samples on tissue microarrays. Both Cohort 1 and Cohort 2 samples were obtained from ESCC patients diagnosed at the Fourth Hospital of Hebei Medical University. Cohort 3 comprised 74 pairs of ESCC and adjacent non‐cancerous tissue samples, along with 32 ESCC tissue samples on tissue microarrays (HEsoS180Su12), purchased from Shanghai Outdo Biotech Company (Shanghai, China). This study was approved by the Ethics Committee of the Fourth Hospital of Hebei Medical University (Approval Number: 2022KS035) and the Ethics Committee of Shanghai Outdo Biotech Company (Approval Number: SHYJS‐BC‐2310001). All participants provided written informed consent in accordance with the Declaration of Helsinki.

### 2.3 Cells and Antibodies

The immortalized esophageal epithelial cell line NE2, as well as the ESCC cell lines KYSE30, KYSE150, KYSE180, KYSE410, KYSE450, and TE‐1, were all acquired from the Type Culture Collection of the Chinese Academy of Sciences (Shanghai, China). They were stored in liquid nitrogen tanks at the Research Center of the Fourth Hospital of Hebei Medical University. ESCC cells were cultured in RPMI1640 media supplemented with 10% fetal bovine serum (Gibco, Invitrogen Corporation, Carlsbad, CA, USA), 100 units/mL penicillin, and 100 μg/mL streptomycin at 37°C with 5% CO_2_. Each cell line used in this study underwent no more than 20 passages, and mycoplasma contamination was tested every 6 months.

The antibodies used in our study were sourced from various suppliers: IFI30, Bax, Bcl‐2, P16, and P21 from Proteintech (Wuhan, China); caspase‐7 (CASP7) and cleaved caspase‐7 from PTM Biolab (Hangzhou, China); Cytc (CYCS, Cytochrome c) from Bioworld (Nanjing, China); phospho‐JNK (P‐JNK), caspase‐3 and cleaved caspase‐3 from CST (Danvers, MA, USA); JNK and HRAS from Wanleibio (Shenyang, China); β‐actin from Absin (Shanghai, China); and Ki67 from Abcam (Cambridge, MA, USA).

### 2.4 IHC Staining

Tissue slides were first incubated in an oven at 65°C for 30 min. After deparaffinization, tissue slices were rehydrated using graded ethanol, treated with 0.3% hydrogen peroxide for 10 min, subjected to heat‐mediated antigen retrieval using citrate (pH 6.0), and finally incubated with primary antibodies overnight at 4°C. Next, the goat anti‐rabbit secondary antibody (PV‐6001; Zhongshan Golden Bridge, Beijing, China) was incubated for half an hour at 37°C. Finally, the DAB substrate solution was applied to visualize positive staining, followed by counterstaining with hematoxylin. Each section was examined and imaged using a microscope (Nikon, Tokyo, Japan). The IHC scores were evaluated by two pathologists in a double‐blind fashion. Total IHC scores were derived by multiplying staining intensity scores (0, none; 1, weak; 2, moderate; 3, strong) with staining percentage scores [1 (1%–25%), 2 (26%–50%), 3 (51%–75%), and 4 (76%–100%)]. Finally, the median IHC score across all tissues was used to determine high or low expression levels of IFI30 and Ki67.

### 2.5 Western Blot Assay

Protease inhibitor‐containing RIPA buffer (Solarbio, China) was used to extract cellular proteins. After being separated on 10% SDS‐PAGE gels, 30 μg of proteins were transferred to PVDF membranes (Millipore, Germany). The membranes were subsequently blocked with TBST solution containing 5% skim milk for 90 min at room temperature. The matched primary antibodies against IFI30 (1:2000), Bax (1:2000), Cytc (1:500), caspase‐7 and cleaved caspase‐7 (1:500), caspase‐3 and cleaved caspase‐3 (1:1000), Bcl‐2 (1:2000), P16 (1:1000), P21 (1:1000), JNK (1:500), P‐JNK (1:1000), HRAS (1:750), and β‐actin (1:5000) were incubated with the membranes overnight at 4°C. The next day, membranes were treated with fluorescent secondary antibodies in the dark for 1 h, and the protein bands were visualized using infrared imaging equipment (LI‐COR, USA). ImageJ software was employed to quantify the intensity of the bands for the target protein and the internal reference protein, β‐actin. The ratio of target protein band intensity to reference protein band β‐actin intensity was calculated, and all data were normalized to the control group, with the control value set to 1 for each experiment.

### 2.6 Short Interfering RNA (siRNA) Transfection

siRNA against IFI30 was synthesized by GenePharma (Suzhou, China). The sequences were as follows: IFI30 siRNA (siIFI30) #1: sense 5′‐GGCCACCAGUUAACUACAATT‐3′ and antisense 5′‐UUGUAGUUAACUGGUGGCCTT‐3′; siIFI30#2: sense 5′‐GCCAGUUGUACCAGGGCAATT‐3′ and antisense 5′‐UUGCCCUGGUACAACUGGCTT‐3′; negative control siRNA (siNC): sense 5′‐UUCUCCGAACGUGUCACGUTT‐3′ and antisense 5′‐ACGUGACACGUUCGGAGAATT‐3′. Lipofectamine 2000 (Invitrogen, USA) was used for siRNA transfection according to the manufacturer's instructions. Cells were harvested for further analysis after 24–72 h of transfection.

### 2.7 RNA Extraction and Reverse Transcription Quantitative Polymerase Chain Reaction (RT‐qPCR) Examination

Total RNA was isolated from cells and tissues using Trizol reagent (Invitrogen, USA). cDNA was synthesized from 2 μg of total RNA using a reverse transcription kit (PRA5001, Promega, USA) and amplified in a Real‐Time PCR System (Bio‐Rad, USA) with GoTaq qPCR Master Mix (A600A, Promega, USA). The 2‐∆∆Ct method was used to calculate the relative gene expression after normalization to GAPDH. The primer sequences were as follows. IFI30: forward: 5′‐TCCAATGCACCGCTTGTCAAT‐3′ and reverse: 5′‐ACCTTGTTGAATTTGCACTCCTC‐3′; mitogen‐activated protein kinase 9 (MAPK9): forward: 5′‐AGACTGCACCCTGAAGATCC‐3′ and reverse: 5′‐TACCGTGTCACCACGTAAGG‐3′; CYCS: forward: 5′‐TGGGCCAAATCTCCATGGTC‐3′ and reverse: 5′‐ATTGGCGGCTGTGTAAGAGT‐3′; VDAC2: forward: 5′‐ACTCTGGGAACAGAAATCGCA‐3′ and reverse: 5′‐CAAGCCAGCCCTCATAACCA‐3′; MRE11: forward: 5′‐ATGCTGTGCTACCTACCCCT‐3′ and reverse: 5′‐TCTCACCCAGACCCACCTAA‐3′; SMAD2: forward: 5′‐ATTTGCTGCTCTTCTGGCTCAG‐3′ and reverse: 5′‐ACTTGTTACCGTCTGCCTTCG‐3′; RRAS2: forward: 5′‐ACAGTTAGCACGGCAGCTT‐3′ and reverse: 5′‐TTTCCGTGTTGGTTCTGGTGA‐3′; HRAS: forward: 5′‐GCAGATCAAACGGGTGAAGG‐3′ and reverse: 5′‐CAGCCAGGTCACACTTGTTC‐3′; IL‐6: forward: 5′‐TGAGGAGACTTGCCTGGTGAA‐3′ and reverse: 5′‐CAGCTCTGGCTTGTTCCTCAC‐3′; IL‐8: forward: 5′‐ATACTCCAAACCTTTCCACCC‐3′ and reverse: 5′‐TCTGCACCCAGTTTTCCTTG‐3′; TNF‐α: forward: 5′‐TATCCTGGGGGACCCAATGT‐3′ and reverse: 5′‐AAAAGAAGGCACAGAGGCCA‐3′; TGF‐β: forward: 5′‐ACCTGCCACAGATCCCCTAT‐3′ and reverse: 5′‐CTCCCGGCAAAAGGTAGGAG‐3′; MMP‐9: forward: 5′‐CGCAGACATCGTCATCCAGT‐3′ and reverse: 5′‐GGACCACAACTCGTCATCGT′; GAPDH: forward: 5′‐CTGGGCTACACTGAGCACC‐3′ and reverse: 5′‐AAGTGGTCGTTGAGGGCAATG‐3′.

### 2.8 MTS Proliferation Assay

The MTS assay was performed to assess tumor cell proliferation according to the manufacturer's instructions. After 24 h of transfection, ESCC cells were seeded in six replicates per group in a 96‐well plate at a density of 2 × 10^3^ cells per well in 200 μL of RPMI 1640 medium with 10% FBS. The cells were incubated for 0, 24, 48, and 72 h. Absorbance at 492 nm was recorded using a FlexA‐200 microplate reader (Allsheng, Hangzhou, China) after incubating each well with 15 μL of MTS solution (Promega, Madison, WI, USA) for 2 h at 37°C in the dark. This process was repeated at each of the required time points.

### 2.9 Colony Formation Assay

1000 logarithmic‐phase cells per well were seeded in a 6‐well plate. For 14 days, the cells were cultured in a saturated, humidified incubator with 5% CO_2_ at 37°C. Every 2–3 days, the culture medium was replaced with fresh medium. After 14 days, the cells were fixed in 4% paraformaldehyde for 30 min before being stained with crystal violet (Solarbio, Beijing, China) for 10 min. The number and size of colonies per well were quantified using ImageJ software.

### 2.10 Transwell Migration and Invasion Assay

Cell invasion and migration were assessed using a 24‐well transwell chamber with 8.0 μm pores (Corning, Canton, NY, USA). In summary, 1 × 10^5^ ESCC cells were seeded in the upper chamber using 200 μL of RPMI 1640 medium without FBS, while 600 μL of medium with 20% FBS was added to the bottom chamber as a chemoattractant. After culturing KYSE150 cells for 24 h and KYSE450 cells for 48 h, the cells were fixed in 4% formaldehyde for 10 min and stained with 1% crystal violet for 10 min at room temperature. Non‐migratory cells on the upper membrane surface were removed with cotton swabs, and cells on the bottom side were counted as migrating cells. The cell invasion assay was performed in the same manner as the cell migration assay, except that the transwell chambers were pre‐coated with 200 mg/mL Matrigel (BD Biosciences, USA) and incubated overnight. Furthermore, images were captured using an inverted microscope (Nikon, Tokyo, Japan), and ImageJ software was used to quantify the number of migratory and invasive cells. All of the data were collected from three distinct experiments.

### 2.11 Wound Healing Assay

Cells from each experimental group were seeded into 6‐well plates for the migration assay. Upon reaching 80% confluence, a 10‐μL pipette tip was used to scratch the bottom of each well. Following three PBS washes to eliminate any floating cells, 2 mL of serum‐free media was then added to each well. After scratching, photos were obtained at the same location under an inverted microscope (Nikon, Tokyo, Japan) at 0, 24, and 48 h. The migratory area was then quantified using ImageJ software.

### 2.12 Lentiviral Infection

GenePharma (Suzhou, China) provided the control lentivirus and IFI30 short hairpin RNAs (IFI30‐shRNA). The sequences for the IFI30 shRNA (shIFI30) and the shRNA control (shNC) were 5′‐GGCCACCAGTTAACTACAA‐3′ and 5′‐TTCTCCGAACGTGTCACGT‐3′, respectively. The KYSE150 cells were infected with shIFI30 and shNC lentiviruses following the manufacturer's instructions (GeneChem, China). An inverted fluorescence microscope (Nikon, Tokyo, Japan) was used to evaluate the lentiviral infection rate 72 h post‐infection, after adding 2 μg/mL puromycin to the medium for selection of infected cells.

### 2.13 Xenograft Model in Nude Mice

Five‐week‐old BALB/c nude mice were purchased from Beijing Huafukang Biotechnology Co. Ltd. (Beijing, China). The project received approval from the Experimental Animal Welfare and Ethics Committee of the Fourth Hospital of Hebei Medical University (Approval Number: IACUC‐4th Hos Hebmu‐20240104) and was conducted in accordance with the international standard, the 3Rs of animal welfare. Subcutaneous injections of KYSE150‐shNC and KYSE150‐shIFI30 cells (5 × 10^6^) were made into each nude mouse's right flank (*n* = 5). After injection, mice were monitored every 2 days, and tumor growth was assessed using the formula *V* = 0.5 × length × width^2^. Seventeen days after injection, the mice were euthanized, and their tumors were excised, measured, weighed, and recorded.

### 2.14 Proteomic Analysis

Proteins were isolated by sonicating KYSE150 cells from the siIFI30#1 and siNC groups in RIPA buffer for 6 cycles (5 s on, 5 s off) and then denatured at 95°C for 2 min. The supernatant was centrifuged at 12 000 *g* for 10 min to remove any insoluble debris. The protein content was measured using a BCA kit (Thermo Fisher Scientific, Waltham, MA, USA). Protein digestion was carried out using filter‐assisted sample preparation. To cleave disulfide bonds, 50 mM DTT was added to 300 mL of UA buffer (0.1 M HCl, 8 M urea, pH 8.5) and incubated at 37°C for 30 min. Peptides were isolated after digestion using centrifugation, lyophilization, and 0.1% FA acidification. Peptide concentrations were quantified using a BCA kit (Thermo Fisher Scientific).

The liquid chromatography‐mass spectrometry/mass spectrometry analyses were conducted as previously described [27]. 1 μg peptide sample was processed through an EasynLC 1200 HPLC system (Thermo Fisher Scientific) and separated via gradient liquid chromatography at 300 nL/min over 90 min. Buffer A consisted of 0.1% (v/v) FA in H_2_O, and buffer B contained 0.1% (v/v) FA in 80% ACN. The gradient settings were as follows: 2%–8% B for 1 min, 8%–28% B for 60 min, 28%–37% B for 14 min, 37%–100% B for 5 min, and 100% B for 10 min. Proteomic analyses were conducted using the Q‐active HF mass spectrometer (Thermo Fisher Scientific). In positive ion mode, the spray voltage was set to 2100 V and the ion transfer tube temperature to 320°C. Profile spectral data were gathered using the Xcalibur program. A full MS1 scan was conducted using an orbital mass analyzer (350–1500 m/z) with a resolution of 60 000 at m/z 200, AGC set to 3e6, and a maximum IT of 20 ms. This was followed by a “top 20” MS2 scan at m/z 200, resolution 15 000, AGC 1e5, and maximum IT 45 ms. The MS2 spectrum's fixed initial mass was set to 110.0 m/z. The separation window was adjusted to 1.6 m/z. The normalized collision energy was 27%, with a dynamic exclusion time of 45 s. Precursors with charges of 1, 8, and greater than 8 were excluded from MS2 analysis.

### 2.15 Database Analysis of MS Data

We performed an analysis of differentially expressed proteins (DEPs) between the siNC and siIFI30#1 groups. DEPs were identified using a threshold of fold change < 0.667 or > 1.5 with a *p*‐value < 0.05. Gene ontology (GO) includes biological process (BP), cellular component (CC), and molecular function (MF). GO enrichment analysis, ROC curve, volcano plot, heat map, and principal component analysis (PCA) were performed using the online bioinformatics platform (https://www.bioinformatics.com.cn). Additionally, Sangerbox (http://sangerbox.com/Signin), an online platform, was used to conduct Kyoto Encyclopedia of Genes and Genomes (KEGG) pathway enrichment analysis.

### 2.16 Flow Cytometry for Apoptosis and Cell Cycle

Apoptosis and cell cycle were analyzed using the PE Annexin V Apoptosis Detection Kit I (BD Biosciences, San Diego, CA, USA). In order to conduct cell apoptosis tests, KYSE150 and KYSE450 cells in the siIFI30#1, siIFI30#2, and siNC groups were transfected with siRNA for 24 h, and then they were treated with 25 and 12.5 μM cisplatin (DDP) for 24 h, respectively. Following that, the cells were collected and centrifuged for minutes at 800 rpm. The cells were rinsed twice with ice‐cold PBS, then resuspended in 100 μL 1× binding buffer. Cells were incubated with 5 μL PE‐Annexin V and 5 μL 7‐aminoactinomycin D (7‐AAD) for 15 min at room temperature in the dark. Finally, 300 μL of 1× binding buffer was subsequently added to each tube. Additionally, during the cell cycle analysis, cells in each experimental group underwent a 24‐h transfection, were washed with ice‐cold PBS, and were fixed overnight in 75% ethanol at −20°C. Subsequently, the cells were incubated with 20 μL of 7‐AAD solution for 30 min in the dark. Cell apoptosis and cell cycle were assessed using the FACSCalibur flow cytometer (BD Biosciences, Franklin Lake, NJ, USA).

### 2.17 SA‐β‐Gal Staining

SA‐β‐gal staining was performed following the manufacturer's protocol using a kit from Beyotime (Shanghai, China). The culture medium was removed from cells in 6‐well plates, followed by a PBS wash and fixation with 1 mL of fixative solution for 15 min at room temperature. Each well was filled with 1 mL of staining solution after three PBS washes. The wells were sealed with parafilm and incubated overnight at 37°C without CO_2_. Next, the cells were examined using an inverted microscope (Nikon, Tokyo, Japan).

### 2.18 Measurement of the Levels of Intracellular ROS

The amount of ROS within cells was examined using fluorescence imaging. 48 h post‐siRNA transfection, cells were incubated with a DCFH‐DA probe (Beyotime, Shanghai, China) at 37°C for 20 min. The cells were rinsed three times with serum‐free media to remove any residual DCFH‐DA. Fluorescence images for each group were captured using an inverted fluorescence microscope (Nikon, Tokyo, Japan). The fluorescence intensities were quantified using ImageJ software (NIH, USA).

### 2.19 Protein–Protein Interaction (PPI) Network Construction and Analysis

The PPI network of DEPs resulting from IFI30 knockdown in ESCC cells was constructed using the STRING database (STRING, version 12.0; https://string‐db.org) [28], and the minimum interaction score was set to 0.4 (medium confidence). The top 10 hub proteins of the PPI network were identified and visualized using the bottleneck algorithm of the CytoHubba plug‐in in Cytoscape software (version 3.7.1) [29].

### 2.20 Statistical Analyses

Data were expressed as means ± SD and analyzed using GraphPad Prism 8.0 software (GraphPad Software Inc., San Diego, CA, USA). Group differences were assessed using the Student's *t*‐test, while multiple group comparisons were performed using one‐way ANOVA. Statistical analysis of the relationship between patients' clinical pathological characteristics and IFI30 expression was performed using SPSS software (version 27.0; IBM, Armonk, NY, USA). The comparison of categorical variables was conducted using the χ2 test or Fisher's exact test. The Kaplan‐Meier method was used to estimate survival probabilities, and the log‐rank test was employed to compare differences between groups. Statistical significance was defined as *p* < 0.05, with specific thresholds indicated as follows: *p < 0.05, **p < 0.01, ***p < 0.001, ****p < 0.0001.

## 3 Results

### 3.1 IFI30 Is Highly Expressed in ESCC and Associated With Poor Prognosis

**FIGURE 1:**
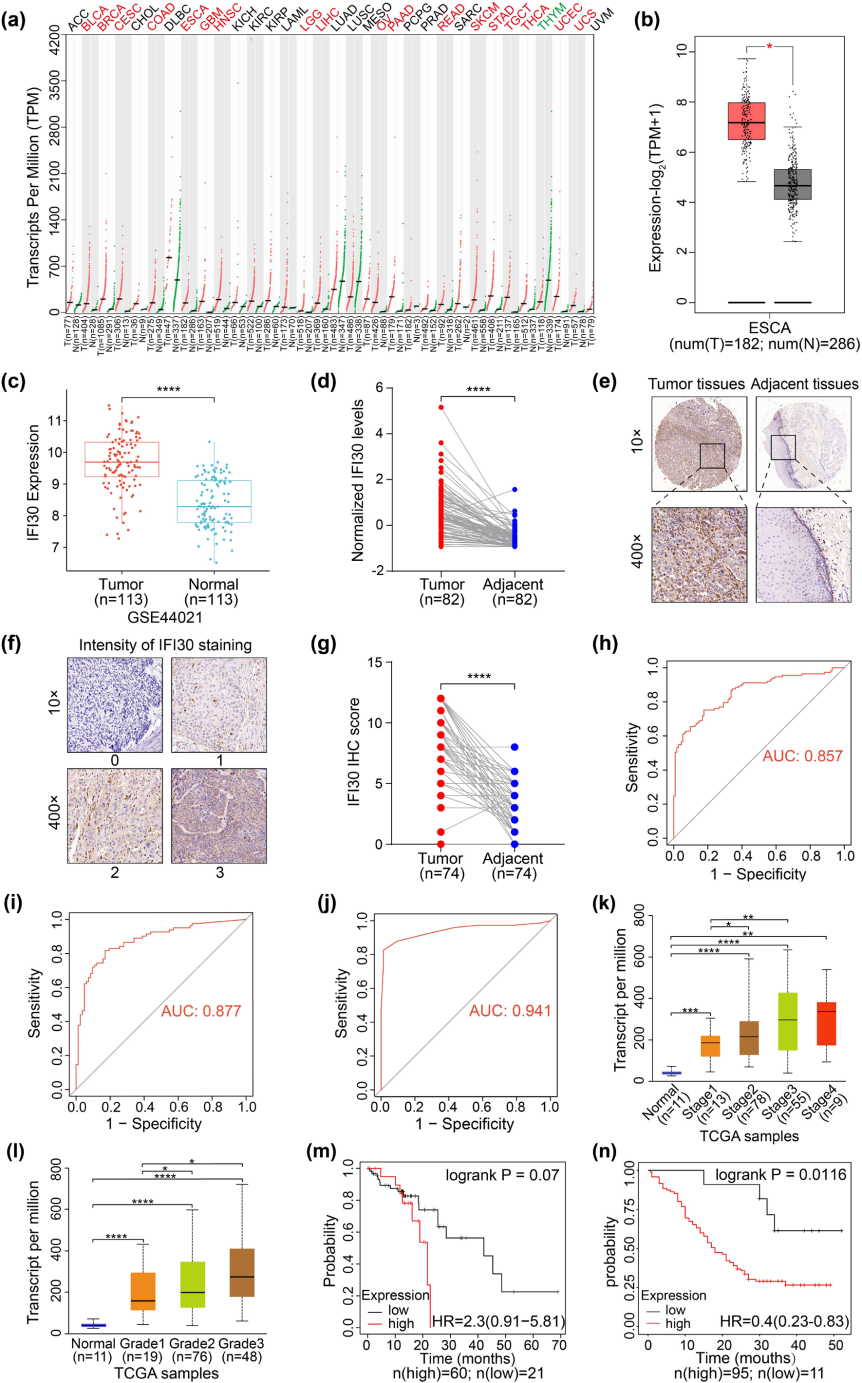
IFI30 is highly expressed in ESCC. (a) The expression profile of the IFI30 gene in all tumor samples and paired normal tissues from the GEPIA2 database. Each point represents the expression level of a sample. Tumor samples are shown in red, and paired normal tissues are shown in green. (b) Comparison of IFI30 mRNA expression levels between ESCC and normal tissues using the GEPIA2 database. Tumor samples are represented in red, and paired normal tissues are represented in gray. (c) IFI30 mRNA expression levels in ESCC and paired normal tissues from the GEO dataset, GSE44021. (d) IFI30 protein expression levels were assessed using proteomics data from 82 pairs of ESCC and adjacent non‐cancerous tissues. Data were standardized using the *z*‐score normalization approach. (e) Representative IHC images of IFI30 expression in tissue microarray HEsoS180Su12. (f) Representative images of IHC staining intensity for IFI30 in tissue microarray HEsoS180Su12. (g) IFI30 protein expression levels in tissue microarray HEsoS180Su12 of 74 pairs of ESCC and adjacent non‐cancerous tissues. (h) ROC curve analysis to assess the diagnostic significance of IFI30 mRNA expression levels in the GEO dataset, GSE44021. (i, j) ROC curve analysis using proteomics data and tissue microarray HEsoS180Su12 data to evaluate the diagnostic value of IFI30 protein expression levels. (k, l) The correlation between IFI30 mRNA expression and individual cancer stage and tumor grade in ESCA was shown using the UALCAN database. (m, n) Kaplan–Meier survival curves for IFI30 expression in ESCC, based on data from the Kaplan–Meier Plotter and tissue microarray HEsoS180Su12. *p < 0.05, **p < 0.01, ****p < 0.0001.

We first analyzed the expression of IFI30 in ESCC tissues. Data from the GEPIA2 database showed that, compared with normal tissues, IFI30 mRNA was generally elevated in 18 different cancers, including ESCA (Figure 1a,b). Further analysis of the GEO database ESCC gene expression profile (GSE44021) revealed that IFI30 mRNA expression was significantly higher in ESCC tissues compared with paired normal tissues (Figure 1c). Since proteins are the functional executors of gene expression, we performed proteomic analysis on 82 pairs of ESCC tissues and adjacent non‐cancerous tissues and found that IFI30 protein expression was significantly higher in ESCC tissues than in paired non‐tumor tissues (Figure 1d). IHC analysis of a tissue microarray containing 41 pairs of ESCC and adjacent non‐cancerous tissues also showed significantly higher levels of IFI30 protein expression (Figure S1a,b). Additionally, IHC analysis of the HEsoS180Su12 tissue microarray further confirmed the increased expression of IFI30 in ESCC tissues (Figure 1e–g).

To evaluate the clinical significance of IFI30 expression in ESCC, we performed ROC curve analysis using the GEO database, which demonstrated that IFI30 mRNA had high diagnostic potential (Figure 1h). Similarly, ROC curves generated from proteomics data and two independent tissue microarray cohorts confirmed the high diagnostic value of the IFI30 protein (Figure 1i,j and Figure S1c). Further analysis using the UALCAN database revealed that higher IFI30 mRNA expression was associated with advanced cancer stages and higher tumor grades in ESCA (Figure 1k,l). The results indicated a trend of negative correlation between IFI30 expression in ESCC samples and OS, although the difference was not statistically significant (*p* = 0.07) (Figure 1m). Analysis of the HEsoS180Su12 tissue microarray further revealed that high IFI30 expression was associated with male patients, later pathological stage, shorter overall survival, and increased mortality (*p* < 0.05) (Table 1, Figure 1n). Overall, these findings highlight IFI30 as a diagnostic and prognostic marker, as well as its potential role as a tumor‐promoting factor in ESCC progression.

**TABLE 1:** The association between clinicopathological features and IFI30 expression in ESCC patients.

Variable	Total (*n* = 106)	IFI30 expression		*χ* ^2^	*p*
		Low (%)	High (%)		
Age (years)				0.031	0.860
< 65	46	4 (36.4)	42 (44.2)		
≥ 65	60	7 (63.6)	53 (55.8)		
Sex				3.895	0.048*
Male	86	6 (54.5)	80 (84.2)		
Female	20	5 (45.5)	15 (15.8)		
Pathological grade				0.260	0.610
I–II	79	7 (63.6)	72 (75.8)		
III	27	4 (36.4)	23 (24.2)		
pT stage				0.000	1.000
T1–T2	17	2 (18.2)	15 (15.8)		
T3–T4	89	9 (81.8)	80 (84.2)		
pN stage				1.391	0.238
N0	45	7 (63.6)	38 (40.0)		
N1–N3	61	4 (36.4)	57 (60.0)		
pTNM stage				4.759	0.029*
I–II	49	9 (81.8)	40 (42.1)		
III–IV	57	2 (18.2)	55 (57.9)		
Survival state				4.111	0.043*
Alive	34	7 (63.6)	27 (28.4)		
Dead	72	4 (36.4)	68 (71.6)		

* Statistical significance *p* < 0.05.

### 3.2 Knocking Down IFI30 Inhibits ESCC Cells Proliferation, Invasion, and Migration

**FIGURE 2:**
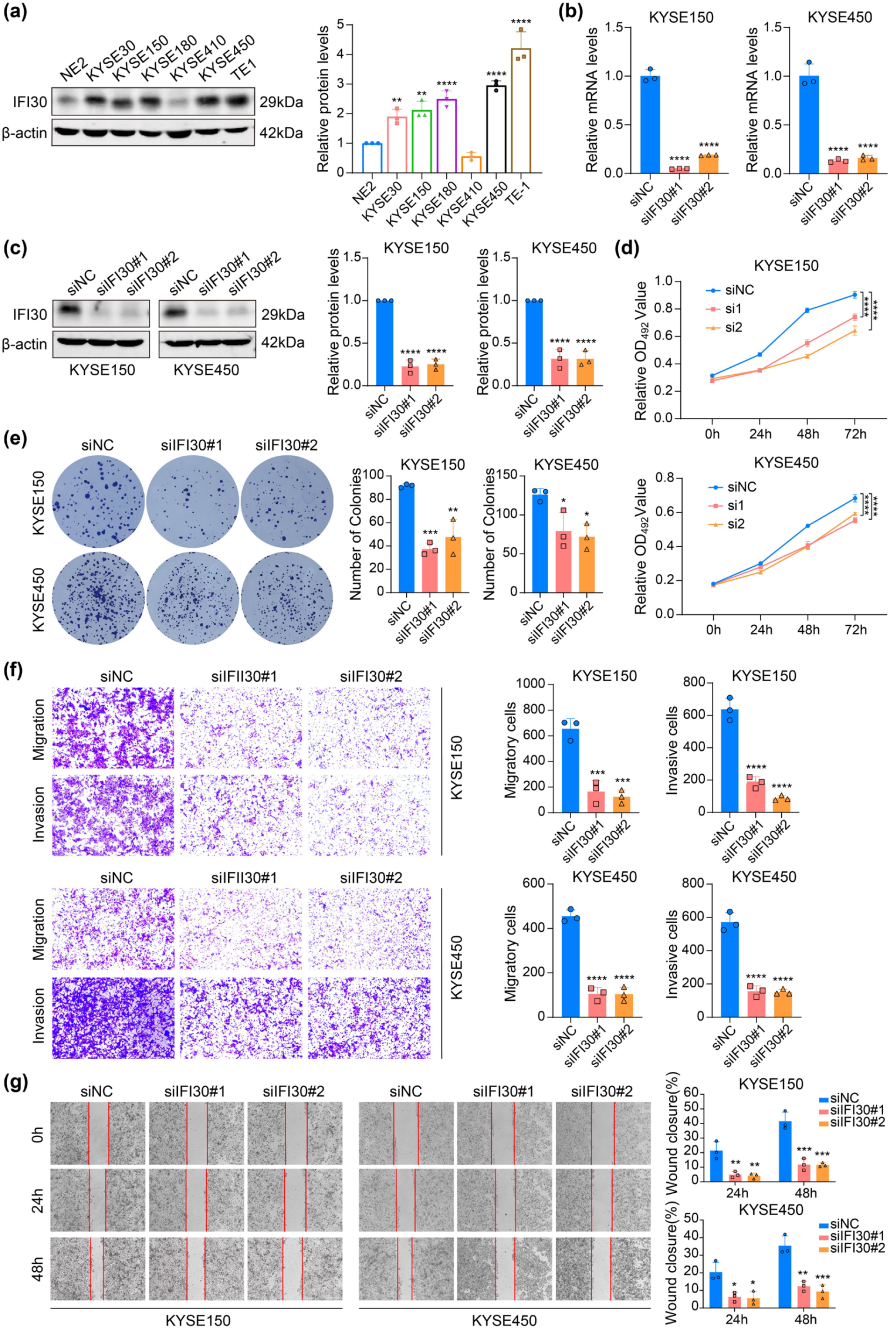
IFI30 knockdown could inhibit ESCC cell proliferation, invasion, and migration. (a) The IFI30 protein expression levels in the esophageal epithelial cell line NE2 and various ESCC cells were assessed using Western blot. (b, c) The knockdown effectiveness of IFI30 in KYSE150 and KYSE450 was validated using RT‐qPCR and Western blot. (d) The growth curve for IFI30 knockdown in KYSE150 and KYSE450 cells was plotted using MTS cell proliferation assay data. (e) The effect of IFI30 knockdown on KYSE150 and KYSE450 cell proliferation was examined using a clone formation assay. (f) The impact of IFI30 knockdown on KYSE150 and KYSE450 cell migration and invasion was assessed using the transwell assay (100× magnification). (g) The impact of IFI30 knockdown on cell migration was evaluated through a wound healing assay (40× magnification). Error bars represent mean ± SD calculated from three biological replicates, each consisting of three technical replicates. *p < 0.05, **p < 0.01, ***p < 0.001, ****p < 0.0001.

To evaluate the biological function of IFI30 in ESCC, Western blot analysis was conducted to evaluate IFI30 protein expression in the immortalized esophageal epithelial cell line NE2 and various human ESCC cell lines (Figure 2a). IFI30 expression was generally higher in the ESCC cell lines compared to the NE2 control. The cell lines KYSE450 and KYSE150 were selected for further investigation due to their higher levels of IFI30 expression. To investigate IFI30's role in promoting ESCC progression, KYSE150 and KYSE450 cells were transiently transfected with siIFI30#1, siIFI30#2, or siNC. The successful knockdown of IFI30 in both KYSE150 and KYSE450 cells was confirmed by RT‐qPCR and Western blot (Figure 2b,c). Subsequently, MTS assays demonstrated that IFI30 knockdown significantly inhibited the growth of KYSE150 and KYSE450 cells (Figure 2d). Similar findings were obtained with the colony formation assay for both KYSE150 and KYSE450 cells (Figure 2e). Next, we utilized the transwell assay to examine the influence of IFI30 on cell migration and invasion. The results showed that IFI30 knockdown decreased the migration and invasion of KYSE150 and KYSE450 cells (Figure 2f). Additionally, wound healing assays confirmed that IFI30 knockdown significantly reduced cell migration (Figure 2g). These findings suggest that decreased IFI30 expression resulted in reduced ESCC cell proliferation, invasion, and migration, highlighting its crucial role in ESCC progression.

### 3.3 Knocking Down IFI30 in ESCC Cells Impairs Tumorigenesis In Vivo

**FIGURE 3:**
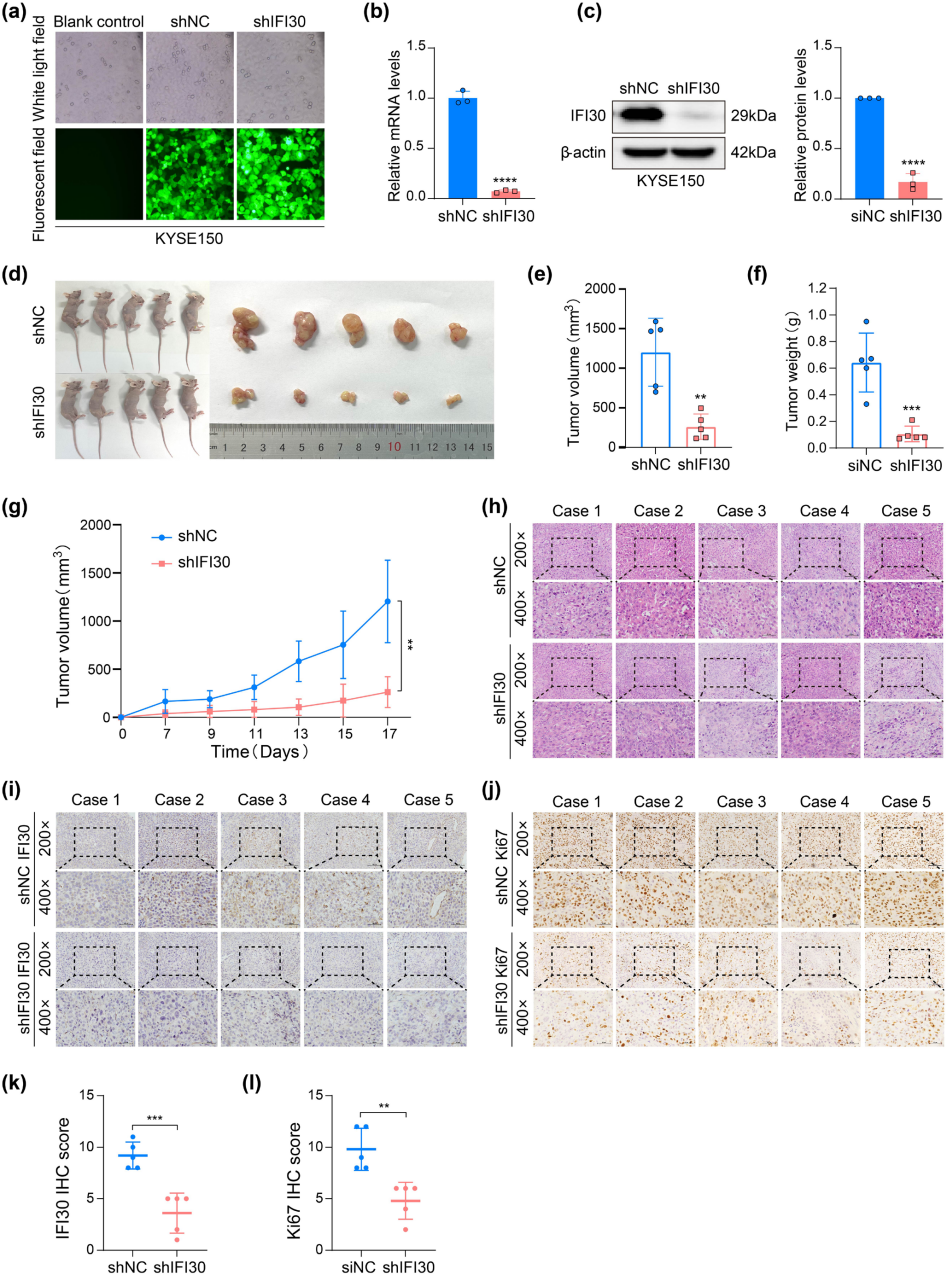
IFI30 knockdown in ESCC impairs tumor growth in vivo. (a) GFP expression was used to assess transfection effectiveness in KYSE150 cells. (b, c) The efficiency of IFI30 knockdown via lentivirus‐mediated shRNA was assessed using RT‐qPCR and Western blot. (d) Images of nude mice and tumors in the shNC and shIFI30 groups. (e, f) The volume and weight of tumors in the shNC and shIFI30 groups were measured after injection. (g) The tumor growth curve in nude mice was plotted by measuring tumor volume at specified intervals. (h) HE staining of tumor tissues in the shNC and shIFI30 groups. (i–l) IHC staining was performed to evaluate IFI30 and Ki67 expression levels in tumor tissues from the shNC and SHFI30 groups. Error bars in (b, c), represent the mean ± SD calculated from three biological replicates, each composed of three technical replicates. The data in (e–g, k, l) are presented as mean ± SD from 5 mice per group. **p < 0.01, ***p < 0.001, ****p < 0.0001.

To further investigate the role of IFI30 in vivo, we performed xenograft model studies in nude mice. First, KYSE150 cells were transfected with either shNC or shIFI30 for 72 h, with transfection efficiency greater than 90%, as validated by fluorescence microscopy of GFP‐positive cells (Figure 3a). RT‐qPCR and Western blot analysis confirmed that the shIFI30 group exhibited significantly lower IFI30 expression compared to the shNC control group (Figure 3b,c). Next, 5 × 10^6^ KYSE150 cells with stable IFI30 knockdown were subcutaneously injected into nude mice, with shNC KYSE150 cells similarly implanted as experimental controls. As anticipated, the results demonstrated that nude mice with IFI30 knockdown exhibited significantly reduced tumor volume and weight compared to controls (Figure 3d–f). The tumor growth curve demonstrated that IFI30 knockdown inhibited tumor growth, which became more evident with time (Figure 3g). HE staining was performed to analyze the pathological morphology of the tumors (Figure 3h). IHC staining revealed significantly lower IFI30 and Ki67 scores in the shIFI30 group compared to the shNC group (Figure 3i–l). These findings indicate that IFI30 knockdown inhibits the tumorigenic activity of ESCC cells in vivo, which is consistent with the in vitro results.

### 3.4 Knocking Down IFI30 Enhances DDP‐Induced Apoptosis and Promotes Cellular Senescence in ESCC Cells

**FIGURE 4:**
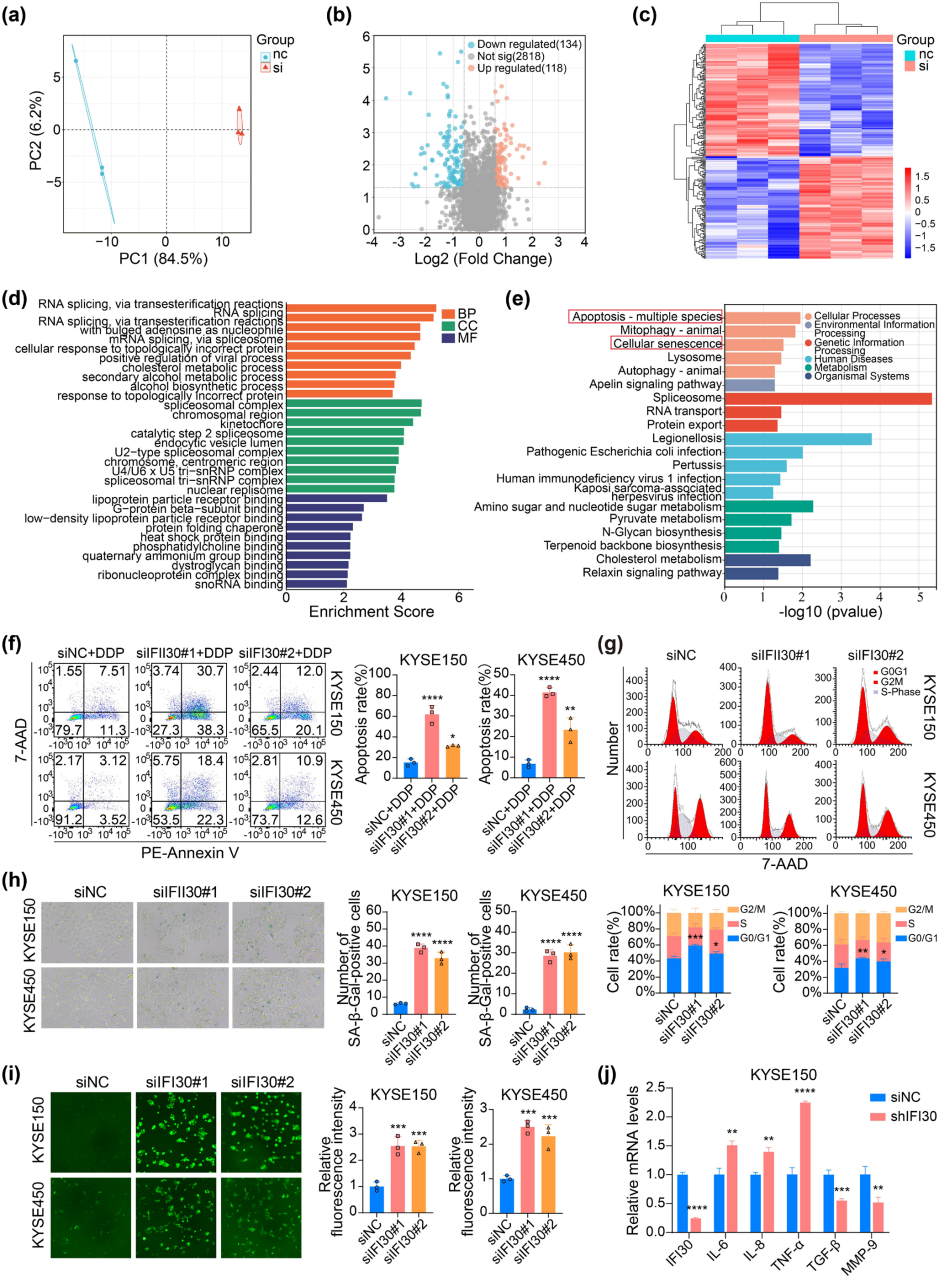
Knocking down IFI30 enhances DDP‐induced apoptosis and promotes cellular senescence in ESCC cells. (a) Principal component analysis of DEPs. (b, c) Volcano plot and heat map of DEPs. (d) GO functional enrichment analysis of DEPs. (e) KEGG functional enrichment analysis of DEPs. (f) Effect of IFI30 knockdown on DDP‐induced apoptosis in KYSE150 and KYSE450 cells, assessed by flow cytometry. (g–i) Impact of IFI30 knockdown on cell cycle progression, senescence‐associated SA‐β‐gal (200× magnification), and ROS production (100× magnification) in KYSE150 and KYSE450 cells. (j) Expression of SASP‐related cytokines and growth factors associated with ESCC progression analyzed by qPCR in KYSE150 cells. Error bars represent mean ± SD calculated from three biological replicates, each consisting of three technical replicates. *p < 0.05, **p < 0.01, ***p < 0.001, ****p < 0.0001.

To investigate the underlying mechanism of IFI30 on ESCC, mass spectrometry‐based proteomic analysis was performed on lysates from KYSE150 cells transfected with siIFI30#1 and siNC control. PCA showed a clear distinction between the two groups (Figure 4a). DEPs were identified based on fold changes > 1.5 or < 0.667 and *p*‐value < 0.05. A total of 252 DEPs were identified, including 118 upregulated and 134 downregulated proteins (Figure 4b,c). GO enrichment analysis showed that these DEPs were involved in biological processes (BP) such as RNA splicing, adenosine as nucleophile, mRNA splicing, and cellular response to topologically incorrect protein. In terms of cellular components (CC), the DEPs were associated with the spliceosomal complex, chromosomal regions, and kinetochore. Molecular function (MF) terms included lipoprotein particle receptor binding, G‐protein beta‐subunit binding, low‐density lipoprotein particle receptor binding, and so forth (Figure 4d). Additionally, KEGG pathway enrichment analysis revealed that DEPs were primarily involved in apoptosis, mitophagy, cellular senescence, and lysosomal pathways following IFI30 knockdown (Figure 4e).

Next, we evaluated the impact of IFI30 knockdown on ESCC cell apoptosis and senescence. Flow cytometry was used to assess the effect of IFI30 knockdown on DDP‐induced apoptosis in KYSE150 and KYSE450 cells. The results indicated that siIFI30 combined with DDP significantly enhanced apoptosis in both KYSE150 and KYSE450 cells compared to controls treated with DDP alone (Figure 4f). We also assessed cellular senescence, characterized by cell enlargement, flattening, elevated SA‐β‐gal activity, cell cycle arrest, and increased ROS production [30]. Morphological examination revealed enlarged and flattened cells in the siIFI30 group compared to controls (Figure S2a). Flow cytometry demonstrated an increased proportion of cells in the G0/G1 phase in the siIFI30 group (Figure 4g). SA‐β‐gal staining demonstrated a higher number of SA‐β‐gal‐positive cells in the siIFI30 group (Figure 4h). Additionally, IFI30 knockdown led to a significant increase in ROS production, as indicated by an increase in DCF fluorescence intensity (Figure 4i). The above results demonstrate that IFI30 knockdown enhances DDP‐induced apoptosis and promotes cellular senescence in ESCC cells.

To further investigate the impact of IFI30 on senescent cells and the cellular microenvironment, we assessed the effect of IFI30 knockdown on the expression of senescence‐associated secretory phenotype (SASP)‐related cytokines and growth factors associated with ESCC progression in KYSE150 and KYSE450 cells using qPCR. qPCR analysis demonstrated that, compared with control cells, IFI30 knockdown significantly upregulated the levels of pro‐inflammatory cytokines IL‐6, IL‐8, and TNF‐α, while downregulating TGF‐β and MMP‐9 (Figure 4j, Figure S2b). These findings suggest that knocking down IFI30 induces a SASP profile characterized by the release of inflammatory cytokines and a reduced capacity for extracellular matrix remodeling.

### 3.5 Exploration of the Downstream Molecular Mechanism of IFI30 Regulating Apoptosis and Senescence in ESCC

**FIGURE 5:**
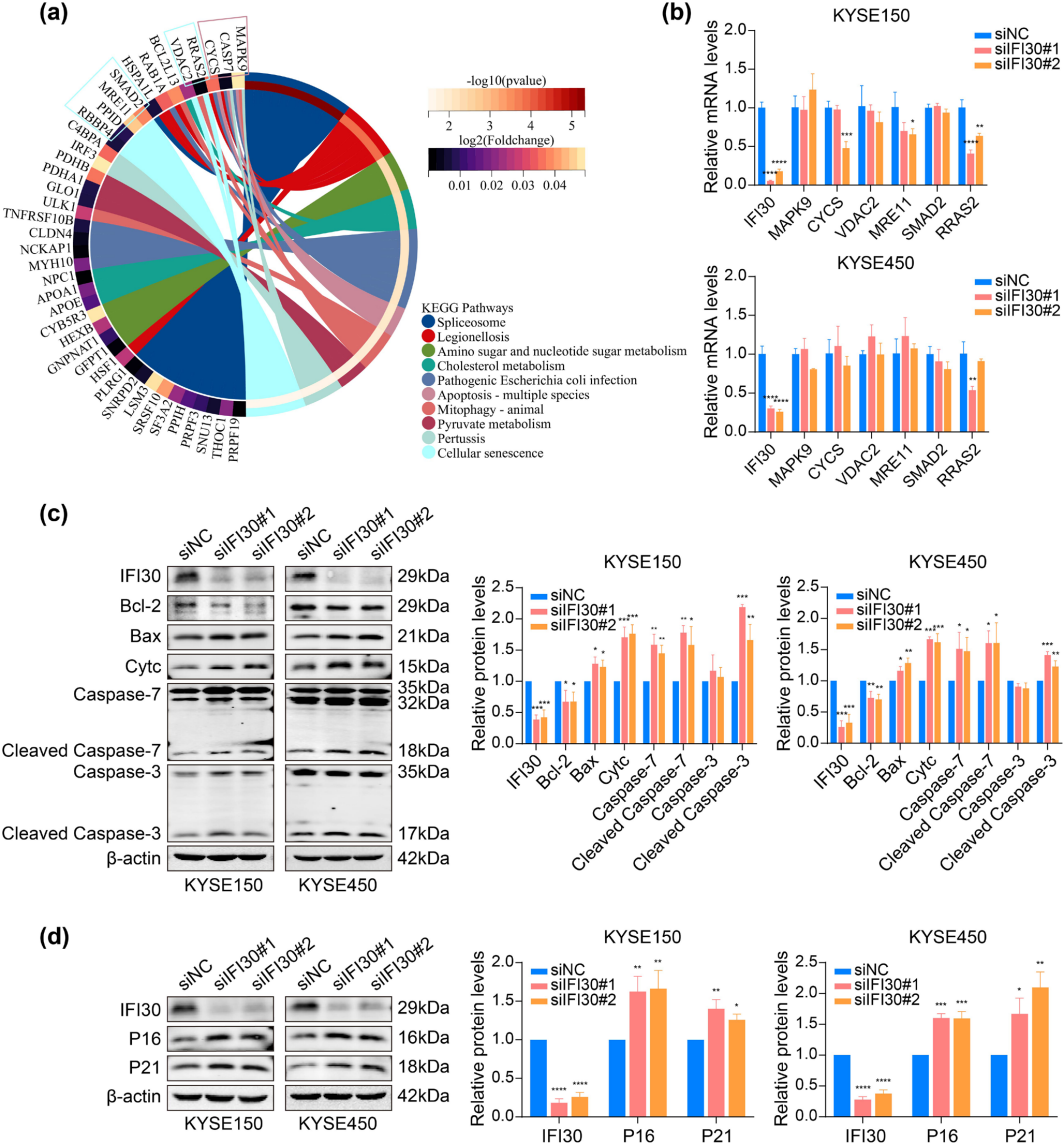
Exploration of the downstream molecular mechanism of IFI30 regulating apoptosis and senescence in ESCC. (a) Chord diagram of the main KEGG pathway for DEPs. (b) RT‐qPCR was used to verify several genes associated with differential proteins in KYSE150 and KYSE450 cells. (c, d) Western blot analysis was performed to measure apoptosis‐associated proteins and senescence‐related proteins following IFI30 knockdown in KYSE150 and KYSE450 cells. Error bars represent mean ± SD calculated from three biological replicates, each consisting of three technical replicates. *p < 0.05, **p < 0.01, ***p < 0.001, ****p < 0.0001.

To investigate the downstream molecular mechanisms through which IFI30 regulates apoptosis and senescence in ESCC, we identified DEPs enriched in apoptosis‐ and senescence‐related pathways (Figure 5a). Several genes encoding DEPs, including MAPK9, CYCS, SMAD2, VDAC2, MRE11, and RRAS2, were selected for RT‐qPCR validation based on a review of the relevant literature. The results showed that CYCS and MRE11 expression levels decreased in the siIFI30#2 group, while RRAS2 was downregulated in both siIFI30#1 and siIFI30#2 groups in KYSE150 cells (Figure 5b). In KYSE450 cells, RRAS2 expression was reduced in the siIFI30#1 group, while other genes showed no significant changes (Figure 5b). These findings suggest that IFI30 may influence the protein expression of these genes except for RRAS2. Next, apoptosis‐associated proteins, including caspase‐7 and Cytc, were examined using Western blot analysis. The results revealed that IFI30 knockdown in KYSE150 and KYSE450 cells increased levels of Bax, Cytc, caspase‐7, cleaved caspase‐7, and cleaved caspase‐3, while decreasing Bcl‐2 levels (Figure 5c), indicating that IFI30 knockdown promotes apoptosis in ESCC cells. Additionally, the expression of senescence markers P16 and P21 was significantly increased following IFI30 knockdown (Figure 5d).

**FIGURE 6:**
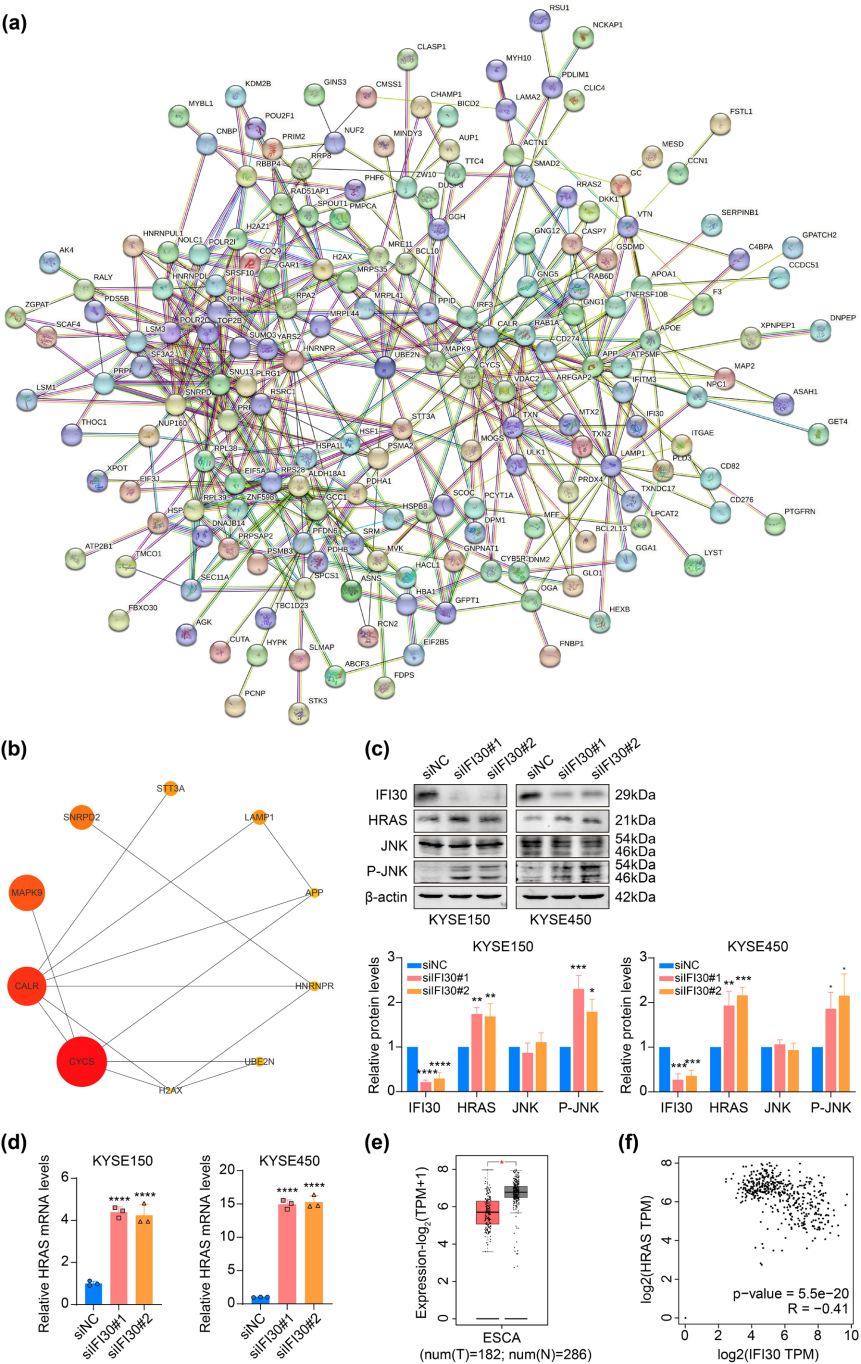
Analysis and validation of the hub proteins of IFI30 regulating apoptosis and senescence in ESCC. (a) The PPI network of DEGs resulting from IFI30 knockdown in ESCC cells. (b) The top 10 hub proteins in the PPI network. (c) The protein expression of the downstream pathways of IFI30 knockdown in KYSE150 and KYSE450 cells was examined by Western blot. (d) The mRNA expression of HRAS in KYSE150 and KYSE450 cells following IFI30 knockdown was detected using RT‐qPCR. (e) HRAS mRNA expression levels in ESCA and normal tissues were assessed using the GEPIA2 database. (f) The GEPIA2 database shows a correlation between the expression of IFI30 and HRAS. Error bars in the bar graph represent the mean ± SD calculated from three biological replicates, each composed of three technical replicates. *p < 0.05, **p < 0.01, ***p < 0.001, ****p < 0.0001.

We further generated a PPI network of DEPs from IFI30 knockdown in ESCC cells using the STRING database and determined the top 10 hub proteins using Cytoscape software, including CYCS, CALR, MAPK9, SNRPD2, LAMP1, STT3A, HNRNPR, APP, UBE2N, and H2AX (Figure 6a,b). Notably, CYCS and MAPK9 were enriched in apoptosis‐related pathways and also served as hub proteins (Figures 5a and 6b). MAPK9 (JNK2), a member of the JNK kinase family, is known for its tumor‐suppressive and apoptotic functions [31]. Our previous experiments have confirmed the expression of CYCS (Figure 5c). Therefore, we further analyzed the levels of JNK and P‐JNK to investigate the impact of IFI30 knockdown on the JNK signaling pathway. As expected, IFI30 knockdown significantly increased JNK phosphorylation without affecting JNK expression levels (Figure 6c). Previous studies have shown that high expression of HRAS induced apoptosis by activating JNK and promoted cellular senescence through the upregulation of P16/P21 [32, 33, 34]. Therefore, we examined the expression of HRAS mRNA and protein using RT‐qPCR and Western blot. The results revealed that both HRAS mRNA and protein expression were upregulated in the siIFI30 group in KYSE150 and KYSE450 cells (Figure 6c,d). Furthermore, bioinformatics analysis of HRAS mRNA expression in ESCA tissues revealed significantly lower expression levels compared to normal tissues (Figure 6e). A moderate negative correlation between IFI30 and HRAS expression was also observed (Figure 6f). Taken together, these results suggested that IFI30 knockdown in ESCC cells resulted in an increase in HRAS expression, activation of the JNK signaling pathway, elevated apoptotic protein levels, and upregulation of P16 and P21.

## 4 Discussion

This study presents the first evidence of the oncogenic function of IFI30 in ESCC. Our data indicate that IFI30 is markedly overexpressed in ESCC tissues and cell lines, with its expression correlating with advanced clinical stages and poor prognostic outcomes. These findings emphasize the potential utility of IFI30 as both a diagnostic biomarker and a prognostic indicator for ESCC. Functional assays revealed that knockdown of IFI30 significantly inhibited cell proliferation, migration, and invasion, and in vivo experiments showed that IFI30 knockdown markedly suppressed tumor growth. These results suggest that IFI30 acts as an oncogene in ESCC. Notably, the role of IFI30 appears to be context‐dependent in various cancer types: in glioma, elevated IFI30 expression is associated with poor prognosis [12, 18], whereas in melanoma and diffuse large B‐cell lymphoma, it correlates with better survival outcomes [7, 13]. These discrepancies likely reflect the tumor‐type‐specific regulation of downstream targets and signaling pathways by IFI30, underscoring the necessity for cancer‐specific investigations.

A significant finding of our study is that knockdown of IFI30 simultaneously induces both apoptosis and cellular senescence, two processes that are pivotal in maintaining tissue homeostasis and inhibiting the abnormal proliferation of damaged or dysfunctional cells [35, 36]. Our data indicate that the knockdown of IFI30 leads to increased levels of pro‐apoptotic proteins Bax, Cytc, cleaved caspase‐7, and cleaved caspase‐3, while concurrently reducing the expression of the anti‐apoptotic protein Bcl‐2. This observation aligns with mitochondrial‐dependent apoptotic mechanisms, which are characterized by an imbalance in Bcl‐2 family proteins. Specifically, the elevated Bax/Bcl‐2 ratio leads to the translocation of Bax to the outer mitochondrial membrane, increasing membrane permeability and facilitating the release of Cytc. The released Cytc interacts with Apaf‐1 and caspase‐9 to form the apoptosome, which subsequently activates caspase‐9, leading to the cleavage and activation of caspase‐3 and caspase‐7, thereby initiating apoptosis [37, 38]. In addition, flow cytometry analysis demonstrated that IFI30 knockdown enhances DDP‐induced apoptosis. A recent study similarly indicated that IFI30 increased resistance to temozolomide, while its knockdown boosts temozolomide‐induced apoptosis in glioma cells. These findings highlight the potential of IFI30 as a target for chemotherapy sensitization.

In addition to apoptosis, our results demonstrate that the knockdown of IFI30 also induces cellular senescence, characterized by increased cell size, a flattened morphology, enhanced SA‐β‐gal activity, G0/G1 phase arrest, and increased ROS production. The upregulation of cyclin‐dependent kinase inhibitors P16 and P21 further confirms the occurrence of senescence. These findings are consistent with the known features of senescence markers [30]. Moreover, the newly induced SASP characteristics by IFI30 knockdown, including elevated levels of IL‐6, IL‐8, and TNF‐α, as well as decreased levels of TGF‐β and MMP‐9, reflect the dynamic interplay between senescence and tumor suppression [30]. While SASP factors such as IL‐6 and TNF‐α may promote immune cell‐mediated clearance of senescent cells, they could also support tumor progression by fostering a pro‐inflammatory microenvironment [30, 39, 40]. In contrast, the downregulation of TGF‐β and MMP‐9 is associated with inhibited matrix remodeling and invasiveness [41, 42], which aligns with our functional experiments demonstrating that IFI30 knockdown contributes to tumor suppression.

The decision‐making process between cellular senescence and apoptosis is complex and is influenced by various factors, including the nature, intensity, and duration of stress, as well as alterations in intracellular signaling pathways [43, 44, 45]. Typically, mild DNA damage induces senescence, whereas severe or prolonged DNA damage results in apoptosis [46, 47]. Similarly, moderate levels of ROS can trigger senescence, while elevated ROS levels promote apoptosis [44, 48]. Moreover, cellular responses to various stress signals leading to apoptosis or senescence involve the regulation of multiple signaling pathways, including the P53, ERK, MAPK, Akt, and NF‐kB pathways [43]. Our research identifies that the JNK signaling pathway may serve as a key mediator in balancing these two cellular fates. Proteomic analysis and subsequent validation experiments revealed that IFI30 knockdown significantly enhanced ROS production and JNK phosphorylation. Previous studies have demonstrated that JNK can induce mitochondrial apoptosis by altering the Bax/Bcl‐2 ratio [49, 50]. Specifically, upon apoptotic stimulation, activated JNK translocates to the mitochondria and phosphorylates Bcl‐2 proteins, thereby antagonizing the anti‐apoptotic functions of Bcl‐2 or Bcl‐XL. Additionally, JNK can activate Bax by promoting Bid cleavage and can also phosphorylate Bim and Bmf to activate Bax and/or Bak [51, 52], thereby initiating mitochondrial‐dependent apoptosis [53]. JNK signaling has also been shown to promote cellular senescence by the upregulation of the p53/p21 and p16/Rb pathways, a process that involves autophagy [54, 55]. Additionally, increased ROS can persistently activate JNK, leading to cell cycle arrest and apoptosis [45]. Therefore, the elevated ROS levels induced by IFI30 knockdown may sustain JNK activation, potentially serving as a key mechanism driving apoptosis and senescence in ESCC cells.

Interestingly, our study also revealed that IFI30 knockdown significantly upregulated HRAS mRNA and protein expression in ESCC cells. This finding is consistent with previous reports indicating that elevated HRAS expression leads to sustained activation of the JNK signaling pathway, accompanied by the inhibition of the ERK pathway, which in turn induces cell apoptosis. BRCA1 has been shown to induce apoptosis in breast and ovarian cancer cells by activating the HRAS‐MEKK4‐JNK‐FasL/Fas‐caspase‐9 cascade. Additionally, overactivation of HRAS can promote cellular senescence through upregulation of the p53/p21 and p16 pathways [32, 33, 34]. Notably, bioinformatics analysis of clinical ESCC samples further confirmed a negative correlation between IFI30 and HRAS expression levels in ESCC tissues. Therefore, we hypothesize that IFI30 knockdown may promote cell apoptosis and senescence by upregulating HRAS, thereby activating the JNK and P21/P16 signaling pathways.

Our findings suggest that IFI30 may be a promising diagnostic and prognostic biomarker, as well as a potential therapeutic target for ESCC. Targeting IFI30 could confer several advantages, including inhibiting tumor progression by inducing cell apoptosis and senescence, enhancing chemotherapy sensitivity (such as promoting DDP‐induced apoptosis), and remodeling the tumor microenvironment (such as reprogramming the SASP). Although our study delineates the HRAS‐JNK‐P21/P16 axis, the precise mechanism through which IFI30 regulates HRAS/JNK remains elusive. Therefore, further rescue experiments are needed to confirm the causal relationship. Furthermore, considering the known role of IFI30 in adaptive immunity [7, 18], its potential immunoregulatory function in ESCC merits further exploration.

In conclusion, IFI30 is highly expressed in ESCC and is associated with advanced stage and poor prognosis. Knockdown of IFI30 in ESCC inhibits cell proliferation, migration, and invasion in vitro, as well as tumor growth in vivo. Further studies suggest that IFI30 knockdown may induce apoptosis and senescence by activating the JNK and P21/P16 pathways, thereby suppressing the malignant progression of ESCC. This study provides a theoretical foundation for the potential application of IFI30 as a diagnostic and prognostic biomarker, as well as a therapeutic target in ESCC.

## Author Contributions

Baoen Shan, Lianmei Zhao, Wenyao Xie, and Sisi Wei conceived and designed the study. Wenyao Xie and Sisi Wei designed and performed the experiments. Zhe Zhang collected the tissue samples. Wenyao Xie, Caiting Feng, and Yuhui Fu analyzed the experimental data. Wenyao Xie, Sisi Wei, and Caiting Feng wrote the manuscript of the article. Suli Dai, Cong Zhang, Lianmei Zhao, and Baoen Shan revised the manuscript. All authors have read and agreed to the published version of the manuscript.

## Conflicts of Interest

The authors declare no conflicts of interest.

## Supporting information


Figure S1.

## Data Availability

All the data can be obtained from the corresponding author upon reasonable request.
