# Correction to “IFI30 Knockdown Inhibits ESCC Progression by Promoting Apoptosis and Senescence via Activation of JNK and P21/P16 Pathways”

**DOI:** 10.1111/1759-7714.70087

**Published:** 2025-05-23

W. Xie, S. Wei, C. Feng, et al., “IFI30 Knockdown Inhibits ESCC Progression by Promoting Apoptosis and Senescence via Activation of JNK and P21/P16 Pathways,” *Thoracic Cancer* 16, no. 7 (2025): e70063, https://doi.org/10.1111/1759‐7714.70063.

Figure 1 has two labeling errors.

1. In Panel 1e, the magnification was incorrectly labeled as “10×”; the correct label should be “100×.”
2. In Panel 1f, the magnification was incorrectly labeled as “10×”; the correct label should be “400×.”

These labeling errors occurred during figure preparation and do not affect the image quality, interpretation, or the scientific conclusions of the study. The corrected figure is provided below.

**FIGURE 1:**
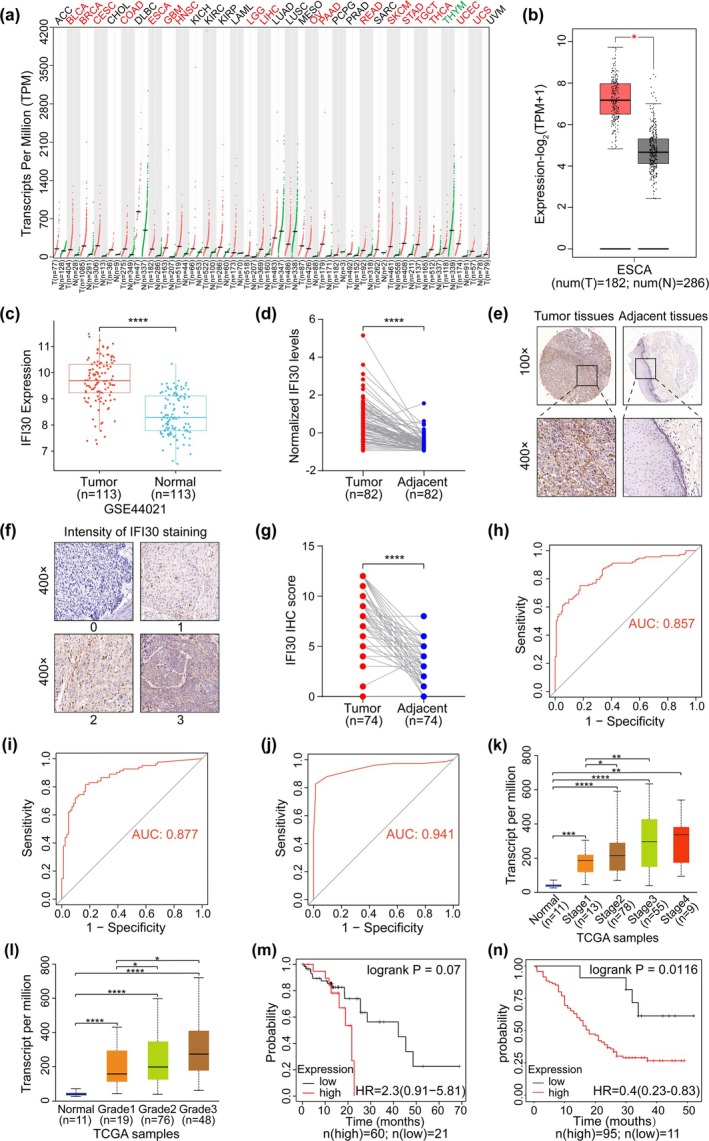
IFI30 is highly expressed in ESCC. (a) The expression profile of the IFI30 gene in all tumor samples and paired normal tissues from the GEPIA2 database. Each point represents the expression level of a sample. Tumor samples are shown in red, and paired normal tissues are shown in green. (b) Comparison of IFI30 mRNA expression levels between ESCC and normal tissues using the GEPIA2 database. Tumor samples are represented in red, and paired normal tissues are represented in gray. (c) IFI30 mRNA expression levels in ESCC and paired normal tissues from the GEO dataset, GSE44021. (d) IFI30 protein expression levels were assessed using proteomics data from 82 pairs of ESCC and adjacent non‐cancerous tissues. Data were standardized using the z‐score normalization approach. (e) Representative IHC images of IFI30 expression in tissue microarray HEsoS180Su12. (f) Representative images of IHC staining intensity for IFI30 in tissue microarray HEsoS180Su12. (g) IFI30 protein expression levels in tissue microarray HEsoS180Su12 of 74 pairs of ESCC and adjacent non‐cancerous tissues. (h) ROC curve analysis to assess the diagnostic significance of IFI30 mRNA expression levels in the GEO dataset, GSE44021. (i, j) ROC curve analysis using proteomics data and tissue microarray HEsoS180Su12 data to evaluate the diagnostic value of IFI30 protein expression levels. (k, l) The correlation between IFI30 mRNA expression and individual cancer stage and tumor grade in ESCA was shown using the UALCAN database. (m, n) Kaplan–Meier survival curves for IFI30 expression in ESCC, based on data from the Kaplan–Meier Plotter and tissue microarray HEsoS180Su12. *p < 0.05, **p < 0.01, ****p < 0.0001.

We apologize for these errors.

