# Supplementary material for: IFI30 Knockdown Inhibits ESCC Progression by Promoting Apoptosis and Senescence via Activation of JNK and P21/P16 Pathways
Source: Thorac Cancer. 2025 Apr 5;16(7):e70063. doi: 10.1111/1759-7714.70063 (PMC11971534; doi:10.1111/1759-7714.70063)
Supplement: Supplementary file 1 — Figure S1. [file TCA-16-e70063-s001.docx]

**FIGURE S1** IFI30 is highly expressed in ESCC.


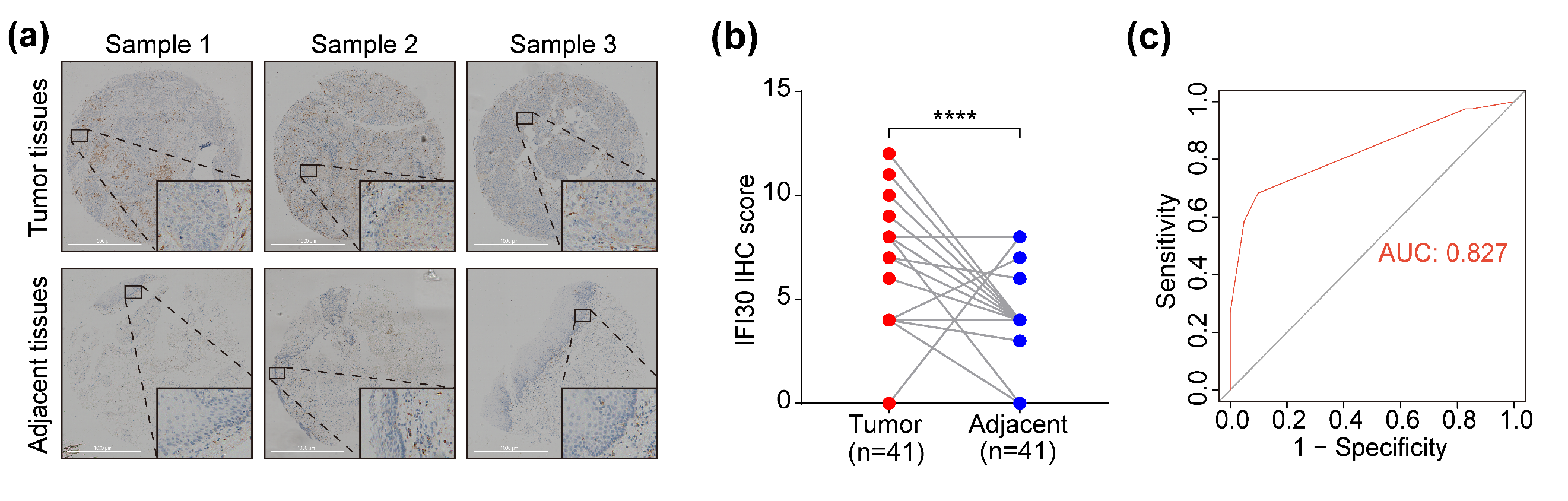


(a) Representative IHC images of IFI30 in tissue microarray of 41 pairs of ESCC and adjacent non-cancerous tissue samples. (b) IFI30 protein expression levels were assessed in tissue microarray of 41 pairs of ESCC and adjacent non-cancerous tissues. (c) ROC curve analysis using a tissue microarray of 41 pairs of ESCC and adjacent non-cancerous tissues to evaluate the diagnostic value of IFI30 protein expression levels.

**FIGURE S2** Knocking down IFI30 promotes cellular senescence in ESCC cells.


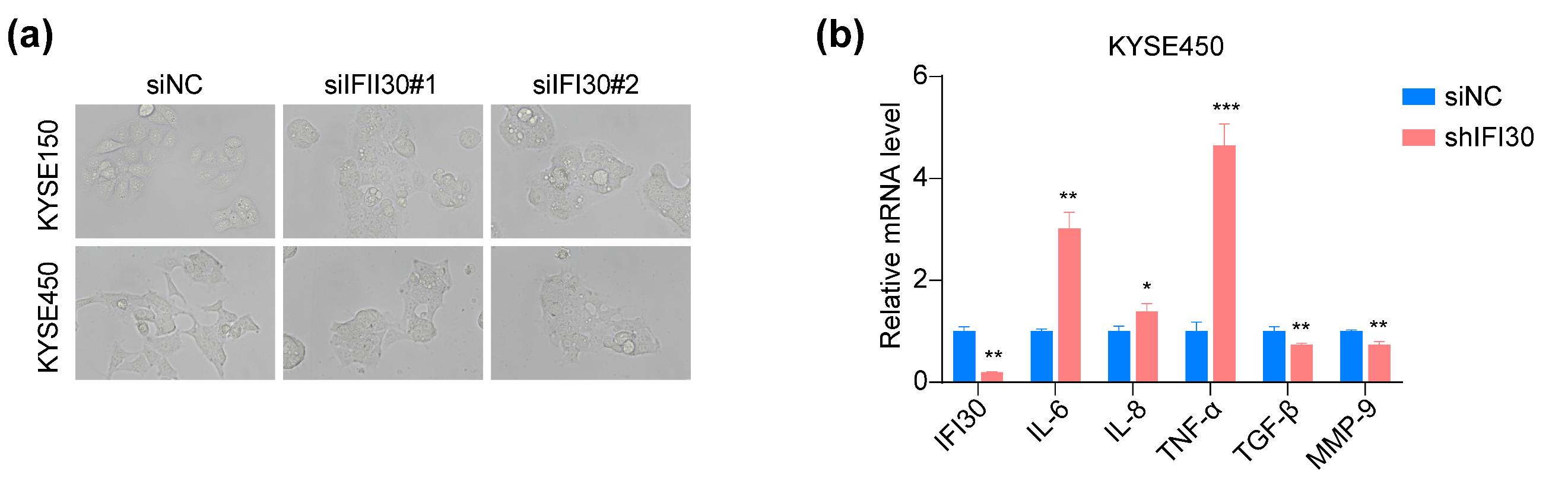


(a) Impact of IFI30 knockdown on morphology in KYSE150 and KYSE450 cells (400× magnification). (b) Expression of SASP-related cytokines and growth factors associated with ESCC progression analyzed by qPCR in KYSE450 cells.
